# A case of adult multisystem Langerhans histiocytosis successfully treated by smoking cessation and radiotherapy for bone lesion

**DOI:** 10.1002/ccr3.6344

**Published:** 2022-09-14

**Authors:** Chie Yamamoto, Taishi Harada, Ryo Sawada, Takumi Sugimoto, Hiroki Hayata

**Affiliations:** ^1^ Department of Respiratory Medicine Fukuchiyama City Hospital Fukuchiyama Japan; ^2^ Department of Oncology Fukuchiyama City Hospital Fukuchiyama Japan; ^3^ Department of Hematology Fukuchiyama City Hospital Fukuchiyama Japan

**Keywords:** adult, Langerhans histiocytosis, multisystem

## Abstract

An adult patient was diagnosed with multisystem Langerhans cell histiocytosis with lung and bone lesions. Her lung lesions improved after smoking cessation. Radiotherapy was performed for the bone lesions. Follow‐up assessment at 2 years after diagnosis showed no recurrence. Our case shows that remission is possible even without systemic treatment.

## Graphical abstract

Even in adult cases of multisystemic Langerhans cell histiocytosis, remission may be achieved without systemic therapy if the symptoms of lung involvement are relieved by smoking cessation.

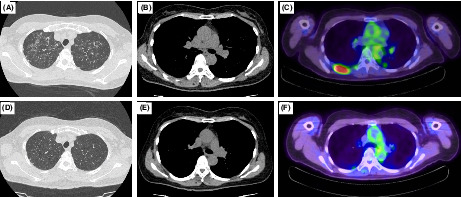

## 1 INTRODUCTION

Langerhans cell histiocytosis (LCH) rarely occurs in adults, and no established treatment has been reported yet.^1^ Smoking cessation is the primary treatment for pulmonary LCH (PLCH).^2^ Systemic therapy is considered for patients who do not respond to smoking cessation, show progression, or present with multisystem LCH (MS‐LCH).^2^ However, there is no standard systemic therapy available yet.^1^, ^3^ We describe the case of an adult patient with MS‐LCH with lung and rib involvement who was successfully treated with smoking cessation and radiotherapy for bone lesions.

## 2 CASE

**FIGURE 1:**
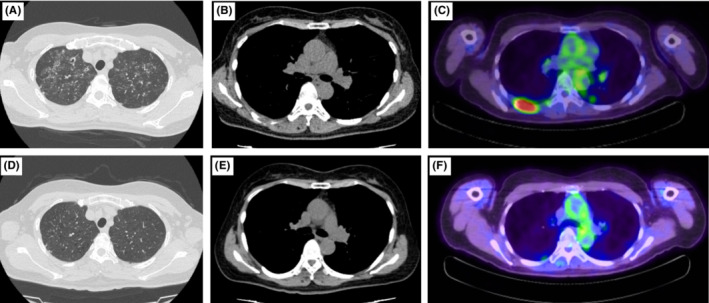
Chest computed tomography (CT) of the (A) lung and (B) bone lesions on Day 1. (C) FDG‐PET/CT on Day 11. CT of the (D) lung and (E) bone lesions as well as (F) FDG‐PET/CT approximately 2 years after the initial visit.

**FIGURE 2:**
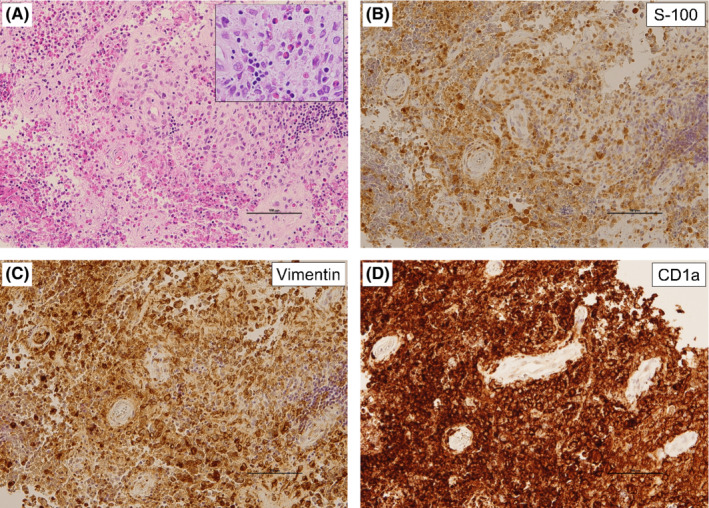
Pathological images with (A) hematoxylin and eosin staining, and (B) S‐100, (C) vimentin, and (D) CD1a immunostaining.

**FIGURE 3:**
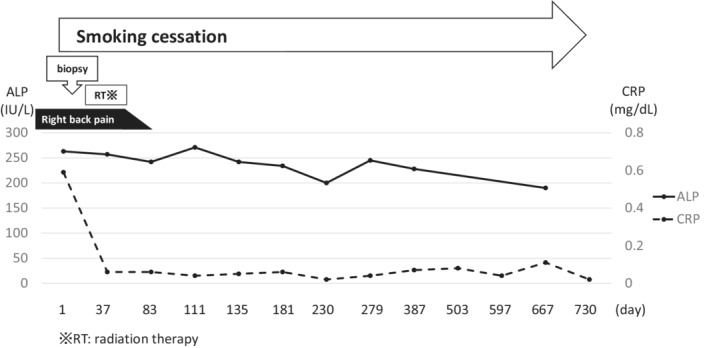
Clinical course of the patient. ※RT, radiation therapy.

The patient was a 60‐year‐old woman whose chief complaint was right back pain. She had a history of uterine fibroids and ovarian cysts, did not consume alcohol, and had smoked approximately 20 cigarettes per day for 40 years from the age of 20 years until cessation of treatment. At the time of initial examination, she had clear consciousness; body temperature, 36.4°C; heart rate, 97 beats/min; respiratory rate, 16 breaths/min; blood pressure, 133/99 mmHg; and oxygen saturation, 96% (room air). Blood tests at the time of initial examination are shown in Table 1, and computed tomography (CT) images are shown in Figures 1A,B. Multiple granular and nodular shadows with cavity formation as well as bronchial wall thickening were observed mainly in both the upper lobes of the lungs, and a nodular lesion with osteolysis was noted on the dorsal side of the right seventh rib. ^18^F‐fluorodeoxyglucose positron emission tomography (FDG‐PET)/CT showed FDG accumulation in the lesions (Figure 1C). Respiratory function tests could not be performed owing to the COVID‐19 outbreak. Transthoracic echocardiography revealed that the tricuspid regurgitation peak gradient was 17 mmHg with no signs of right heart failure. An ultrasound‐guided percutaneous needle biopsy was performed for the lesion on the seventh rib. Hematoxylin and eosin staining revealed a lesion with numerous eosinophilic infiltrates and necrosis as well as scattered large cells with internal bundled nuclei. Immunostaining was positive for the expression of S‐100, vimentin, and CD1a (Figures 2A–D), leading to the diagnosis of LCH. The BRAF‐V600 mutation analysis (BML) of the same specimen had positive results. A bronchoalveolar lavage was performed on the right upper lobe B1aii, but the recovery rate was only approximately 12% and no significant cytological results were obtained. Both smear and culture test results of the bronchoalveolar lavage fluid were negative for common bacteria and acid‐fast bacilli. Considering the above findings, the patient was diagnosed with MS‐LCH with lung and bone involvement. The clinical course of the patient is shown in Figure 3. Smoking cessation was initiated, and the patient underwent palliative radiotherapy at 20 Gy/10 times for the lesion on the right seventh rib owing to severe pain even after administration of painkillers. After radiotherapy completion, systemic therapy was considered, but since the lung lesion improved markedly after smoking cessation and no new lesions were observed in other parts of her body, smoking cessation alone was decided on as follow‐up treatment. For approximately 2 years since the initial diagnosis, neither lung lesions nor bone lesions relapsed, and no new lesions were observed. Figures 1D–F show the CT and FDG‐PET/CT images obtained 2 years after the initial diagnosis.

**TABLE 1:** Laboratory findings on Day 1

WBC	8690 (3500–9100)	/μl	TP	7.6 (6.7–8.3)	g/dl	CEA	2.4 (0.0–5.0)	ng/ml
Neutro	59.5 (42.0–74.0)	%	ALB	4.4 (3.8–5.5)	g/dl	CYFRA	1.0 (0.0–3.5)	ng/ml
Lymph	31.5 (18.0–50.0)	%	AST	23 (0–40)	IU/L	ProGRP	42.4 (0–81.0)	pg/ml
Mono	6.2 (1.0–8.0)	%	ALT	22 (0–45)	IU/L	sIL‐2R	443 (121–613)	U/ml
Eosino	2.1 (0.0–7.0)	%	LDH	228 (115–245)	IU/L	ACE	11.0 (8.3–21.4)	U/L
Baso	0.3 (0.0–2.0)	%	ALP	263 (110–360)	IU/L	Lyzozyme	7.6 (5.0–10.2)	μg/ml
RBC	429 × 10^4^ (376–500)	/μl	γ‐GTP	24 (0–45)	IU/L	β‐D glucan	19.9 (0.0–20.0)	pg/ml
Hb	13.7 (11.3–15.2)	g/dl	BUN	12 (8–22)	mg/dl	T‐SPOT.TB®	(−)	
Ht	41.3 (34–45)	%	Cre	0.54 (0.47–0.79)	mg/dl			
PLT	30.7 × 10^4^ (13.0–36.9)	/μl	Na	141 (138–145)	mEq/L			
			K	4.1 (3.6–4.8)	mEq/L			
			Cl	103 (101–108)	mEq/L			
			CRP	0.59 (0.00–0.30)	mg/dl			

*Note*: ※(): Normal range.

*Abbreviation*: Ht, hematocrit; PLT, pletelet.

## 3 DISCUSSION

Langerhans cell histiocytosis is a disease characterized by aggregation of CD1a+/CD207+ cells and inflammatory cell infiltration. Although the etiology is unknown, the tumor is considered to result from activation of the mitogen‐activated protein kinase pathway and defined as an inflammatory bone marrow tumor in the Histiocyte Society's 2016 revised classification.^1^ The incidence of LCH in adults is estimated to be 1–2 per million,^4^ making it less common than that in children, and there is no standard treatment strategy for LCH in adults. PLCH is the most common form of the disease in adults and is strongly associated with smoking, as approximately 90% of the patients smoke; thus, smoking cessation is the first recommended treatment for PLCH, and the symptoms of many patients are relieved by smoking cessation alone.^5^, ^6^ Systemic therapy is considered for patients who do not respond to smoking cessation, show progression, and present with MS‐LCH.^2^ Corticosteroid, vinblastine, cladribine, cytarabine, methotrexate, doxorubicin, cyclosporine, bleomycin, and BRAF‐inhibitors have been used in systemic therapy, but their efficacies are not certain yet, and some of these drugs are highly toxic.^1^ Moreover, an international registry study stated that despite no treatment for 31% of adult patients with MS‐LCH, the 5‐year survival rate was 91.7%.^7^ Further, there are multiple reports of adult patients with MS‐LCH who have gone into remission by smoking cessation alone or by smoking cessation and local therapy.^8^, ^9^, ^10^, ^11^ It is controversial whether systemic treatment should be administered even in cases of MS‐LCH. Radiation therapy has been reported to be highly effective and safe in adult LCH,^12^, ^13^, ^14^ performed mainly in cases with bone lesions and other soft tissue lesions (including unifocal and multifocal) with pain and other symptoms, high surgical risk, and clinical instability.^1^, ^3^, ^12^ However, there are opinions that local therapy including radiotherapy is not recommended for vertebral lesions with intraspinal and craniofacial bone lesions with soft tissue extensions (orbit, mastoid, sphenoid, or temporal bone) owing to anatomical concerns about damage to the central nervous system; caution is thus required.^3^ The present case was MS‐LCH in an adult with lung and rib involvement. Since the patient had a good understanding of the disease and was able to quit smoking promptly after diagnosis and the lung lesions resolved early after smoking cessation, she only underwent radiotherapy for the bone lesions as palliative treatment of symptoms and was then monitored. As a result, 2 years after the diagnosis, the pulmonary lesions had almost completely disappeared, and there was no recurrence of bone lesions or at other sites.

## 4 CONCLUSIONS

We experienced a case of MS‐LCH in an adult with lung and bone involvement in whom the symptoms of pulmonary lesions were rapidly relieved by smoking cessation and other lesions were treated locally. Patients with such a condition may experience relief of symptoms, even without undergoing systemic therapy.

## AUTHOR CONTRIBUTIONS

Chie Yamamoto served as the author of the report. Taishi Harada, Ryo Sawada, Takumi Sugimoto, and Hiroki Hayata provided significant revisions to the report.

## FUNDING INFORMATION

No funding was received for this work.

## CONFLICT OF INTEREST

The authors declare no conflicts of interest associated with this manuscript.

## ETHICAL APPROVAL

This study was conducted according to the principles of the Declaration of Helsinki.

## CONSENT

Patient consent was obtained for the publication of this report and accompanying images.

## Data Availability

None.
